# Expression of myxovirus‐resistance protein A: a possible marker of muscle disease activity and autoantibody specificities in juvenile dermatomyositis

**DOI:** 10.1111/nan.12498

**Published:** 2018-06-04

**Authors:** S. Soponkanaporn, C. T. Deakin, P. W. Schutz, L. R. Marshall, S. A. Yasin, C. M. Johnson, E. Sag, S. L. Tansley, N. J. McHugh, L. R. Wedderburn, T. S. Jacques

**Affiliations:** ^1^ Infection, Immunity and Inflammation Programme UCL Great Ormond Street Institute of Child Health London UK; ^2^ Division of Rheumatology Department of Pediatrics Faculty of Medicine Ramathibodi Hospital Mahidol University Bangkok Thailand; ^3^ Division of Neuropathology Vancouver General Hospital Vancouver BC Canada; ^4^ Department of Pathology University of British Columbia Vancouver BC Canada; ^5^ Department of Pediatric Rheumatology Faculty of Medicine Hacettepe University Ankara Turkey; ^6^ Department of Pharmacy and Pharmacology University of Bath Bath UK; ^7^ Rheumatology Unit Great Ormond Street Hospital for Children London UK; ^8^ NIHR Biomedical Research Centre at Great Ormond Street Hospital for Children NHS Foundation Trust and University College London London UK; ^9^ Arthritis Research UK Centre for Adolescent Rheumatology at UCL, UCLH and GOSH London UK; ^10^ Developmental Biology and Cancer Programme UCL Great Ormond Street Institute of Child Health London UK

**Keywords:** biopsy, disease activity, interferon, juvenile dermatomyositis, myositis, myositis‐specific autoantibody

## Abstract

**Aims:**

To evaluate the relationship between expression of myxovirus‐resistance protein A (MxA) protein on muscle biopsies by immunohistochemistry and disease activity in juvenile dermatomyositis (JDM) patients. Also, another aim was to investigate whether the expression of MxA is related with myositis‐specific autoantibodies (MSA) status in JDM patients.

**Methods:**

103 patients (median aged 6.3, interquartile range 0.5–15.9) enrolled in the Juvenile Dermatomyositis Cohort and Biomarker Study (JDCBS). Muscle biopsies were stained with MxA and scored. Clinical data at initial presentation were collected and autoantibodies were analysed. Multiple linear regression analysis was performed to estimate the association between MxA expression on muscle fibres and muscle disease activity, and MSA status.

**Results:**

Expression of MxA protein on JDM samples was identified in 61.2%. There was a significant association between MxA scores and Childhood Myositis Assessment Scale (CMAS) (*P* = 0.002), and Manual Muscle Testing of Eight Muscles (MMT8) (*P* = 0.026). CMAS and MMT8 scores were significantly lower in the group of patients with strong MxA expression. MxA scores differed according to MSA subgroups (*P* = 0.002). Patients with positive nuclear matrix protein 2 autoantibodies had strong MxA expression, whereas anti‐melanoma differentiation‐associated gene 5 positive patients had no or weak MxA expression.

**Conclusions:**

This study reveals the significant association between level of MxA expression on muscle fibres and clinical measures of muscular disease activity in JDM patients and MSA status. This confirms type I interferonopathies in muscle fibres of JDM patients which could help with improving treatment outcome in JDM patients and underscoring the distinct pathophysiological pathways in different MSA status.

## Introduction

Juvenile dermatomyositis (JDM) is a rare chronic inflammatory myopathy of childhood, with primary symptoms including symmetrical, proximal muscle weakness and typical skin rashes including Gottron's papules and heliotrope rash. Activated type I interferon (IFN) pathway has been shown to be a key factor in pathogenesis of JDM and adult DM 1, 2, 3. Myxovirus‐resistance protein A (MxA) which is one of the type I IFN‐induced proteins, and is specifically regulated by the type I IFN pathway 4, 5. During the last decade, type I IFN gene expression in blood has been shown to correlate with disease activity and muscle involvement in DM patients 6, 7. Also, MxA expression in muscle of DM patients has been previously demonstrated 2, 8. Moreover, a previous report has shown that the expression of type I IFN signature genes in muscle tissues of adult DM patients was stronger than in JDM patients except for MX1 gene 9. Since muscle is the major target of inflammation in JDM, the presence of MxA protein in muscle biopsies could be of relevance to direct mechanisms of tissue injury. This study aimed to evaluate the relationship between expression of MxA protein on muscle biopsies by immunohistochemistry (IHC) and disease activity in JDM patients. Moreover, myositis‐specific autoantibodies (MSA) which are exclusively present in myositis patients, have been shown to help describe distinct groups of patients with similar clinical characteristics, treatment outcome and prognosis 10, 11, 12, 13, 14, 15. A secondary aim of this study was to investigate whether the expression of MxA is related with MSA status in JDM patients.

## Materials and methods

### Patients, clinical features and laboratory data

This study included clinical data and muscle biopsy samples from 103 patients with definite or probable JDM 16, 17 who were already recruited to the Juvenile Dermatomyositis Cohort and Biomarker Study (JDCBS) 18. Written informed consent and age appropriate assent were received from parents and patients respectively. The study was approved by the Northern & Yorkshire Medical Research and Ethics Committee (MREC), UK. Patient data were obtained through the JDCBS database including age at disease onset, gender, time between onset and biopsy date, and whether any medication was received before muscle biopsy. Clinical features at presentation were collected including the presence of calcinosis, nail fold capillary abnormality, pulmonary involvement, Childhood Myositis Assessment Scale (CMAS) 19, Manual Muscle Testing of Eight Muscles (MMT8) 20, and the physician's global assessment (PGA). The CMAS has a range from 0 to 52, with high scores corresponding to no physical disability. The MMT8 ranges from 0 to 80, with high scores indicating no muscle weakness. The PGA is the assessment of the patient's overall disease activity scored by a physician at the time of assessment, ranging from 0 to 10 (high scores corresponding to maximal disease). Serum levels of muscle enzymes including creatine kinase (CK), lactate dehydrogenase (LDH) and aldolase were routinely collected at the first visit.

### Autoantibody detection

Autoantibodies were screened for by radio‐immunoprecipitation as described previously 21. Anti‐nuclear matrix protein 2 (NXP‐2) and anti‐melanoma differentiation‐associated gene 5 (MDA5) antibodies were subsequently confirmed by enzyme‐linked immunosorbent assay 11, 12. We included patients with MSA and no‐detectable autoantibodies during the analyses in order to assess the relationships between MSA status and MxA expression on JDM muscle specimens.

### Muscle biopsy tissues and scoring data

Open biopsy material obtained from vastus lateralis of 103 patients was analysed. All muscle biopsy samples were snap‐frozen within 1 h of operation and stored at −80°C. Cryostat sections (7 μm) were cut, air‐dried overnight, fixed with acetone, and stored at −80°C until used. All cases had been stained and scored according to a standardized scoring system as described 22, 23. The scoring tool consists of four domains: inflammatory, vascular, muscle fibre and connective tissue domain. Total biopsy score is the sum of all four domains (range 0–27; high scores indicate severe pathological abnormalities). This tool also includes a histopathologists' visual analogue score (hVAS) which assessed the overall severity (range 0–10; high scores indicate worse pathology). A set of normal muscle biopsies from subjects with no muscle disease were analysed in parallel for comparison 24. Biopsy scores and clinical and serology data for most of the patients reported in the present study overlap with those previously reported by our group 25. The MxA staining data have not yet been reported.

### Immunohistochemical staining of MxA protein and microscopic evaluation

A total of 103 patients had muscle biopsy tissues available which were manually stained for MxA by IHC staining. All specimens were also stained and scored using the standardized score tool for JDM histopathology previously reported to maintain consistency 22. Eighteen control muscle biopsy samples from subjects with no muscle disease and no abnormality at light microscopy were included for analysis in comparison. The primary antibodies used to detect MxA protein were mouse anti‐human anti‐MxA monoclonal antibodies (clone: M143, 1:400 dilution; Merck, Kenilworth, NJ, USA) Horseradish peroxidase and DAB (Dako, Cambridgeshire, UK) were used as secondary antibodies and the chromogen respectively. Fiji imaging software (Bethesda, Maryland, USA) was used to process the images. The expression of MxA in muscle fibres was scored semi‐quantitatively by two independent investigators including a senior neurohistopathologist who were blinded to patients' clinical features and laboratory data. The scores ranged from 0 to 3 (0 = no MxA staining; 1 = weak; 2 = moderate; 3 = strong). In each specimen, the patterns of MxA staining were evaluated as either perifascicular, nonperifascicular or both. Perifascicular pattern was defined by the presence of brown staining at the peripheral area of muscle fascicles and the definition of nonperifascicular pattern was patchy or diffuse staining in a section.

### Statistical analyses

Data were analysed using SPSS Statistics for Windows (Version 23.0. Armonk, NY, USA; IBM Corporation). Descriptive statistics of baseline characteristics were summarized using median and interquartile range for numeric variables and percentages for categorical variables. Differences in CMAS scores, MMT8, PGA and serum levels of muscle enzymes between different MxA scoring data were tested by Kruskal–Wallis analysis of variance. *Post hoc* tests were performed to explore significant differences between pairs of MxA scoring data and *P*‐values were adjusted by using Bonferroni's correction for multiple comparisons. Fisher's exact test was used to examine differences of MxA scoring data in different MSA subgroups. To estimate the association between the extent of MxA expression on muscle fibres and disease activity, multiple linear regression analysis was performed. The strength of the association from the regression analysis was described by the standardized coefficient (β). The interobserver variability was analysed using Bland–Altman method which the mean difference (−0.05) and 95% limits of agreement (0.048, −0.148) were calculated and confirmed high agreement. *P*‐values <0.05 were considered statistically significant.

## Results

### Demographic data, clinical features and laboratory data

Demographic data and clinical characteristics of all 103 JDM patients are summarized in Table 1. There was a female predominance (64.1%). Median age at disease onset was 6.3 (0.5–15.9) years and median duration from disease onset to muscle biopsy was 3.8 (2.6–8.7) months. Patients represented a range of disease severities. About 9% and 11% of patients had received corticosteroids and methotrexate at time of muscle biopsy respectively. Since immunosuppressive drugs, such as corticosteroids and methotrexate, have been shown to have an effect on the type 1 IFN pathway 26, we compared patients who had received no drugs prior to biopsy to those who had received immunosuppressive drugs: there were no differences in clinical features, laboratory data and MxA scoring data between the two groups (Table S1). Thus, all 103 patients were included in the study. The three most prevalent MSAs detected in these patients were anti‐transcriptional intermediary factor 1‐gamma (19.8%), anti‐NXP‐2 (18.8%) and anti‐MDA5 (11.9%) which was consistent with previous reports 10, 27.

**Table 1:** Demographic data and clinical features of 103 juvenile dermatomyositis patients at initial presentation

Characteristics	Median (IQR)^a^
Female, *n* (%)	66 (64.1)
Age at disease onset, years	6.3 (0.5–15.9)
Duration from disease onset to first visit, months	4.3 (2.7–9.8)
Time from disease onset to muscle biopsy, months	3.8 (2.6–8.7)
Time from first visit to muscle biopsy, months	0.67 (0.35–0.86)
On immunosuppressive drugs at time of biopsy, *n* (%)	
Corticosteroids	9 (8.7)
Methotrexate	11 (10.7)
CMAS (*n* = 90)	28.5 (16–45)
MMT8 (*n* = 62)	54 (35–71)
PGA (*n* = 72)	5.95 (3.5–7.7)
CK, units/l (*n* = 96)	367 (77–2146.5)
Myositis autoantibodies, *n* (%) (*n* = 101)	
MDA5	12 (11.9)
NXP‐2	19 (18.8)
Mi2	5 (5)
TIF1γ	20 (19.8)
No detectable	19 (18.8)

IQR, interquartile range; CK, creatine kinase; CMAS, Childhood Myositis Assessment Scale (scores 0–52); MDA5, melanoma differentiation‐associated gene 5; MMT8, Manual Muscle Testing of Eight Muscles (scores 0–80); NXP‐2, nuclear matrix protein 2; PGA, physician global assessment (scores 0–10); TIF1γ, transcriptional intermediary factor 1‐gamma.

a Data are presented as median (IQR) if not stated otherwise.

### Expression of MxA protein in muscle fibres from JDM patients

**Figure 1:**
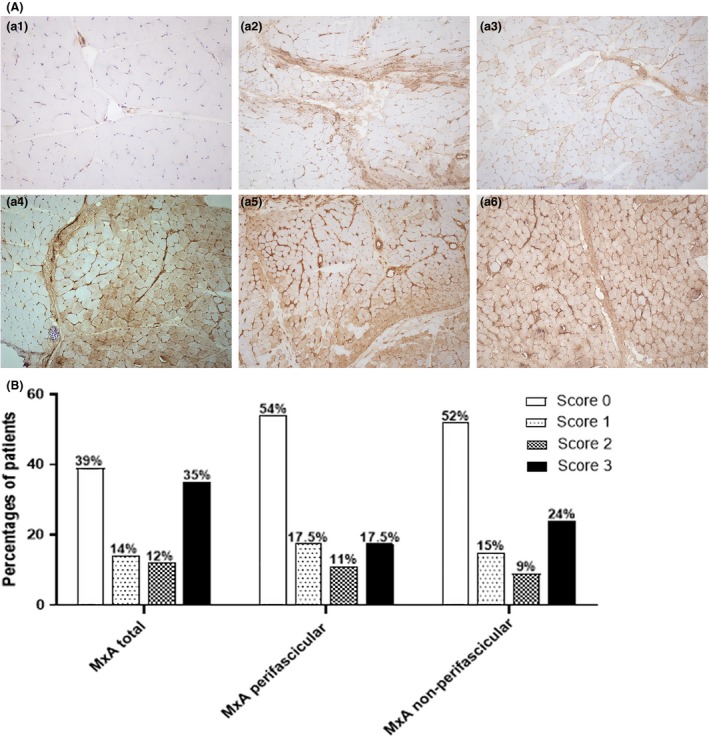
Immunohistochemical staining of myxovirus‐resistance protein A (MxA) in juvenile dermatomyositis (JDM) muscle tissues. (**A**) Representative immunohistochemical staining of MxA in JDM biopsies. (a1) Negative MxA staining in muscle fibres. (a2) Perifascicular MxA expression with score of 1. (a3) Nonperifascicular MxA expression with score of 1. (a4) Nonperifascicular and perifascicular MxA expression with score of 2. (a5) Perifascicular MxA expression with score of 2. (a6) Strong nonperifascicular MxA expression with score of 3. Original magnifications: × 20 (a1); × 10 (a2–6). (**B**) Percentage of patients in different degrees of MxA expression in 103 JDM patients. MxA total score was analysed in a whole image of specimens. In each specimen, the patterns of MxA staining were evaluated as either perifascicular, nonperifascicular or both.

Representative immunohistochemical staining of MxA protein expression in muscle fibres from JDM patients is shown in Figure 1 **A**. Expression of MxA protein on muscle fibres of JDM samples was identified in 63 patients (61.2%) (Figure 1 **B**), whereas 82.5% of JDM patients had diffuse staining on capillaries. Moreover, in 18 normal control muscle biopsies, 67% had capillary staining of MxA while there was no MxA protein expression on muscle fibres in any normal specimens (data not shown). Among JDM patients with positive MxA staining on muscle fibres, more than half (57.1%) had strong MxA expression. The distribution of MxA expression was observed in both perifascicular (46%) and nonperifascicular (53%) patterns.

### Increased MxA expression on muscle fibres was associated with increased muscular disease activity in JDM

**Figure 2:**
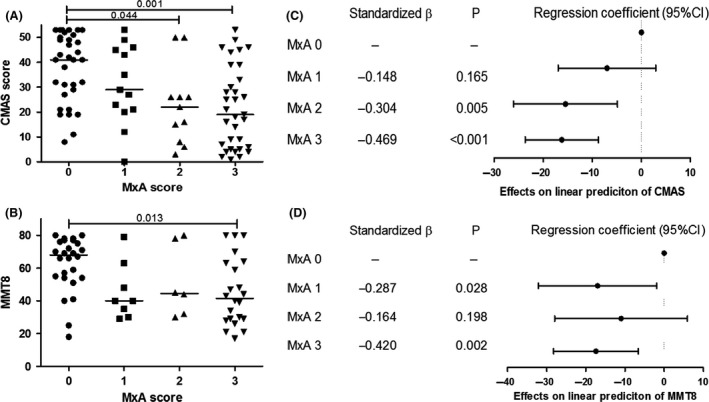
Association between muscular disease activity and relative MxA protein expression on JDM muscle samples. Distribution of (**A**) CMAS score (*P* = 0.002), (**B**) MMT8 (*P* = 0.026) across MxA scoring data. Kruskal–Wallis anova was tested to analyse the difference in the distribution. Horizontal bars show median values. *Post hoc* comparisons were automatically performed when the *P*‐value was statistically significant. Forest plots of linear regression‐estimated coefficients for (**C**) CMAS and (**D**) MMT8 showing significant relationships between MxA protein expression on JDM muscles and muscular disease activity. CMAS, Childhood Myositis Assessment Scale; JDM, juvenile dermatomyositis; MMT8, Manual Muscle Testing of Eight Muscles; MxA, myxovirus resistance protein A.

The distribution of different MxA scores did not differ according to age at disease onset, gender or clinical features at first presentation, such as, the presence of calcinosis, nail fold capillary changes or PGA (Table S2). However, there was a significant association between MxA scores and CMAS, and MMT8 (*P* = 0.002 and 0.026, respectively). The *post hoc* analysis showed the significant differences in CMAS score between patients with MxA scores of 0 and 2 (*P* = 0.044), and MxA scores of 0 and 3 (*P* = 0.001) (Figure 2 **A**). The median CMAS score in the strong MxA expression group was 19 (9–46), whereas in the group with no MxA expression, the CMAS score was 41.5 (29–52). Similarly, MMT8 scores differed significantly between patients with MxA scores of 0 and 3 (*P* = 0.013) (Figure 2 **B**). Since there was evidence of a significant association with time from disease onset to muscle biopsy (*P* = 0.046), multiple linear regression analysis was performed in order to confirm the associations between MxA scoring data and CMAS scores, and MMT8 after adjustment for any confounding effects of time from disease onset to biopsy. From this analysis, expression of MxA protein was found to be significantly associated with CMAS scores and MMT8 at disease onset (Figure 2 **C**,**D**). Thus, patients with MxA scores of 3 had on the average 16 units lower scores CMAS scores when compared to patients with MxA scores of 0 and patients with MxA scores of 1 had on average 7 units lower CMAS scores when compared to patients with MxA scores of 0. Similarly, patients with MxA scores of 3 were associated with an average of 17 units lower MMT8 scores than patients with MxA scores of 0.

Laboratory markers of disease severity, such as CK and LDH were also investigated for the relationship of MxA scoring data. However, serum levels of muscle enzymes were not associated with the expression of MxA protein on JDM muscle samples (Table S2).

### NXP‐2 and MDA5 autoantibodies had differing levels of MxA expression on JDM muscle fibres

**Figure 3:**
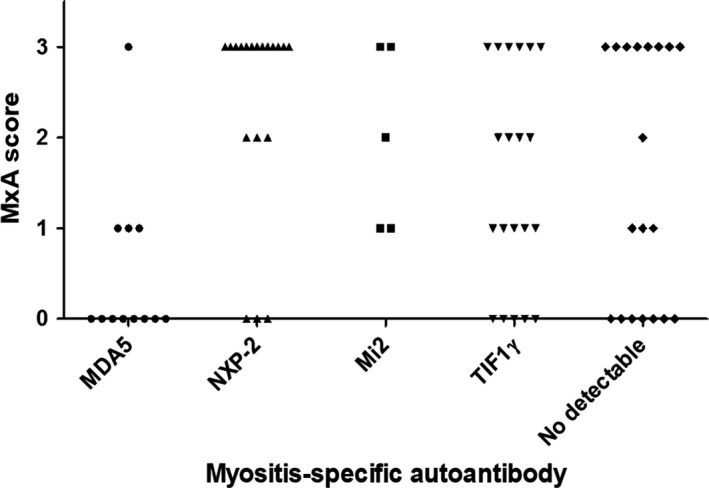
Distributions of MxA protein expression on JDM muscle samples across MSA subgroups. Fisher's exact test was done to analyse the difference in the distributions and *P‐*value was 0.002. JDM, juvenile dermatomyositis; MDA5, melanoma differentiation‐associated gene 5; MSA, myositis‐specific autoantibody; MxA, myxovirus‐resistance protein A; NXP‐2, nuclear matrix protein 2; TIF1γ, transcriptional intermediary factor 1‐gamma.

The extent of MxA expression differed significantly according to MSA subgroups (*P* = 0.002). Patients with positive NXP‐2 autoantibodies tended to have strong MxA expression, whereas anti‐MDA5 positive patients had no or weak MxA expression on muscle biopsies (Figure 3). There was no clear trend in patients with other MSA subtypes or no detectable autoantibodies. According to the distribution patterns of MxA staining, there was no clear association between different MSA subgroups and characteristic MxA stains in JDM muscle samples (*P* = 0.084, Table S3). However, this borderline insignificant finding needs to be further investigated in a larger study.

### The expression of MxA protein on JDM muscle fibres was highly associated with scores of histopathological severity

**Figure 4:**
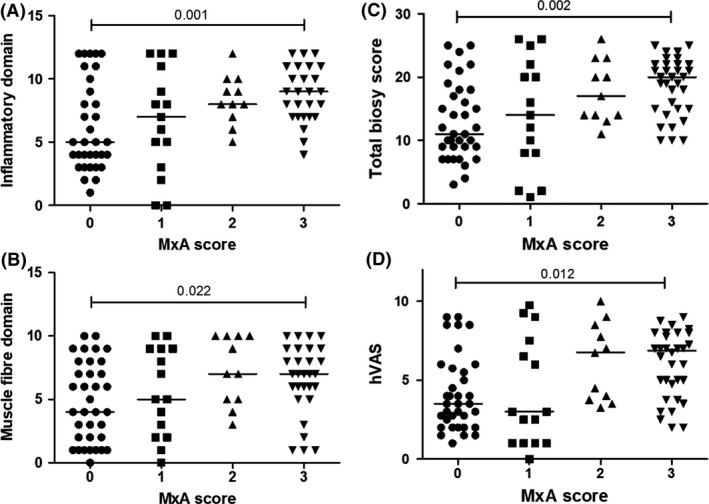
Relationships between MxA scoring data and different domains in a biopsy scoring tool. Distribution of scores in (**A**) inflammatory domain (*P*‐value from Kruskal–Wallis anova was 0.001), (**B**) muscle fibre domain (*P*‐value was 0.014), in a biopsy scoring tool, (**C**) total biopsy score (*P*‐value was 0.002), (**D**) hVAS (*P*‐value was 0.004) in different MxA protein expression on JDM muscle samples. Horizontal bars show median values. *Post hoc* comparisons were automatically performed when the *P*‐value from Kruskal–Wallis anova was statistically significant. hVAS, histopathologist's visual analogue scale global pathology score; IFN, interferon; JDM, juvenile dermatomyositis; MxA, myxovirus‐resistance protein A.

The levels of MxA expression on muscle samples had a positive correlation with several features of the biopsy scoring tool including the inflammatory domain and muscle fibre domains, total biopsy scores and hVAS (Figure 4 **A**–**D**). There were significant statistical differences in those biopsy domains between patients with MxA score of 0 and 3. Interestingly, patients with MxA scores of 0 and 1 tended to have wider range of scores in inflammatory domain, and total biopsy score when compared to patients with MxA scores of 2 and 3.

## Discussion

To our knowledge, this is the first study to demonstrate MxA protein expression on muscle fibres in a large cohort of JDM muscle biopsies (*n* = 103). Assessment muscle biopsies from JDM patients has the potential to reveal pathophysiology of JDM. The expression of MxA protein on muscle biopsies detected by immunohistochemical staining has been recently demonstrated and a role as a marker for diagnosis of DM suggested, in a study of 34 cases of DM (of which 10 were JDM patients) 28. Although the sample size in the previous study was limited, there was about 90% of JDM patients who had positive MxA staining in muscle fibres. The percentages of positive MxA staining in JDM in the previous study is higher than in this study which was about 61% could be explained by the different classification criteria for including of participants and different MxA clones for IHC detection. Further possible explanations would be the differences in median age at disease onset, duration from disease onset to muscle biopsy and the location of muscle the biopsy was taken from. However, these details were not provided in the previous study. Building upon this, our study has demonstrated that MxA expression may also be used as a histological biomarker for JDM disease activity within the muscle tissue. We revealed the strong association between type I IFN response in muscle tissues as assessed by immunohistochemical staining of MxA protein and muscle disease activity in JDM patients. In patients with a high degree of muscle disease severity, MxA protein expression on muscle fibres was found at elevated levels. However, no association was identified between degree of MxA expression on muscle tissue and serum levels of muscle enzymes CK and LDH. These data support that serum levels of muscle enzymes do not correlate well with JDM disease activity as previously reported 29, 30. Previous studies have used gene expression profiling to demonstrate correlation between type I IFN response in blood and muscle disease activity in adult DM and JDM 6, 7, 26, 31. Muscle from adult DM patients showed significant higher levels of MxA expression than in blood 26. This was explained by the hypothesis that some infiltrating inflammatory cells in muscle tissues themselves can express MxA. However, in our study, we measured degrees of MxA protein expression only in muscle fibres, not in inflammatory cells.

Additionally, this is the first study revealing the association of level of MxA protein expression on muscle fibres and MSA subtype specificities in JDM patients. There are few studies showing the correlation between specificities of myositis autoantibodies and IFN pathway activation in blood. A previous study in adult patients showed a significantly higher whole blood IFN score in patients with idiopathic inflammatory myopathies who had anti‐Jo1, anti‐Ro60 and anti‐U1RNP autoantibodies when compared to patients with positive anti‐Ro52 and anti‐PM/Scl 32. However, no data demonstrates the association between other major MSA and levels of IFN pathway activation in JDM patients. Moreover, there are limited studies describing characteristics of muscle histopathology and degrees of muscular inflammation in distinct MSA subtypes. A recent study showed that anti‐NXP‐2 JDM cases had variable scores of histopathological severity 25, whereas a study of adult DM demonstrated that anti‐NXP‐2 patients had less muscle inflammation 33. In the present study, we observed that patients with NXP‐2 autoantibodies had greater degree of muscle inflammation since they had higher level of MxA expression on muscle fibres than other MSA subgroups. In contrast, there was no or low levels of MxA protein expression on muscle biopsies in patients with anti‐MDA5 autoantibodies. Previous studies also showed that anti‐MDA5 antibodies were associated with milder muscle disease activity and muscle pathology than patients with other MSA subgroups 12, 25. Also, adult patients with anti‐MDA5 autoantibodies did not have perifascicular atrophy which is one of the classic pathological features of DM 34, whereas about 58.3% of anti‐MDA5 positive patients in this study had perifascicular atrophy. This could be due to the different pathological findings between adult and paediatric patients. Moreover, a previous Japanese study showed that among muscle biopsies from three patients with anti‐MDA5 autoantibodies, none of the patients had perifascicular atrophy and two patients had MxA expression on muscle fibres 28. In this study, although sample size in this MSA subgroup was limited, about 33.3% of anti‐MDA5 autoantibodies positive patients demonstrated expression of MxA protein on muscle fibres. Another Japanese study demonstrated that anti‐MDA5 autoantibody positive DM patients had higher levels of serum IFN‐α than in those negative for anti‐MDA5 autoantibodies 35. These variable results may reflect genetic and environmental differences between anti‐MDA5 positive patients. It is known that the MDA5 positive patients typically have mild muscle disease and it is therefore possible that IFN inducible proteins mainly circulate in blood but are not highly expressed in muscles of patients with MDA5 autoantibodies. Moreover, some evidence suggests a possible protective mechanism through nitric oxide synthase 2 expression in skeletal muscle of anti‐MDA5 autoantibody positive patients 34. Thus, MDA5 positive patients have limited muscular involvement while they have more severe extramuscular manifestations, such as pulmonary involvement. These results confirm the divergent disease pathophysiology of JDM in different MSA positive groups. Our study showed strong correlations between the degree of MxA expression on muscle fibres and several domains in the JDM biopsy scoring tool 22. Interestingly, the distribution of scores in inflammatory domain and total biopsy scores in patients with no or weak MxA expression tended to have wider range of scores. This may suggest that muscle biopsy specimens with high MxA protein expression might provide more convincing information of muscle tissue injury.

In summary, in this study, we identified MxA protein expression in muscle fibres as a useful histological biomarker of JDM disease activity to supplement the assessment of IFN pathway activation and management of JDM patients. This could help with improving treatment outcome and minimizing comorbidities associated with treatment and disease in JDM patients. Moreover, we emphasize the association of different MSA subgroups in stimulation of the IFN type I pathway which involved in muscle damage in JDM and underscore the distinct pathophysiological pathways in different MSA status. This needs the careful phenotyping of JDM patients for tailored therapy for better outcome.

## Author contributions

SS and LRW designed the studies. LRW and TJ supervised the work. SS, SAY and LRM performed the experiments, PWS, SAY and ES performed the biopsy scores. SLT and NJM generated the MSA data. SS analysed the data and wrote the manuscript. LRM and CMJ analysed patient information. CTD, TSJ and LRW reviewed the manuscript and revised the manuscript. All authors read and approved the final manuscript.

## Conflict of interest

The authors have declared that no conflict of interest exists. The Editors of *Neuropathology and Applied Neurobiology* are committed to peer‐review integrity and upholding the highest standards of review. As such, this article was peer‐reviewed by independent, anonymous expert referees and the authors had no role in either the editorial decision or the handling of the paper.

## Supporting information


**Table S1.** Clinical features and laboratory data at initial presentation between juvenile dermatomyositis (JDM) patients who had received corticosteroid and/or methotrexate and those who had no drugs prior to muscle biopsy.
**Table S2.** Characteristics and laboratory data at initial presentation of 103 juvenile dermatomyositis (JDM) patients in different myxovirus resistance protein A (MxA) scoring data.
**Table S3.** Distributions of patterns of myxovirus resistance protein A (MxA) staining on juvenile dermatomyositis (JDM) muscle samples across myositis‐specific autoantibody (MSA) subgroups*.

## References

[1] Baechler EC , Bilgic H , Reed AM . Type I interferon pathway in adult and juvenile dermatomyositis. Arthritis Res Ther 2011; 13: 249 22192711 10.1186/ar3531 PMC3334651

[2] Greenberg SA , Pinkus JL , Pinkus GS , Burleson T , Sanoudou D , Tawil R , Barohn RJ , Saperstein DS , Briemberg HR , Ericsson M , Park P . Interferon‐alpha/beta‐mediated innate immune mechanisms in dermatomyositis. Ann Neurol 2005; 57: 664–78 15852401 10.1002/ana.20464

[3] Rice GI , Melki I , Frémond ML , Briggs TA , Rodero MP , Kitabayashi N , Oojageer A , Bader‐Meunier B , Belot A , Bodemer C , Quartier P . Assessment of type I interferon signaling in pediatric inflammatory disease. J Clin Immunol 2017; 37: 123–32 27943079 10.1007/s10875-016-0359-1 PMC5325846

[4] Holzinger D , Jorns C , Stertz S , Boisson‐Dupuis S , Thimme R , Weidmann M , Casanova JL , Haller O , Kochs G . Induction of MxA gene expression by influenza A virus requires type I or type III interferon signaling. J Virol 2007; 81: 7776–85 17494065 10.1128/JVI.00546-06 PMC1933351

[5] De Paepe B . Interferons as components of the complex web of reactions sustaining inflammation in idiopathic inflammatory myopathies. Cytokine 2015; 74: 81–7 25481647 10.1016/j.cyto.2014.10.012

[6] O'Connor KA , Abbott KA , Sabin B , Kuroda M , Pachman LM . MxA gene expression in juvenile dermatomyositis peripheral blood mononuclear cells: association with muscle involvement. Clin Immunol 2006; 120: 319–25 16859997 10.1016/j.clim.2006.05.011 PMC3163162

[7] Greenberg SA , Higgs BW , Morehouse C , Walsh RJ , Kong SW , Brohawn P , Zhu W , Amato A , Salajegheh M , White B , Kiener PA . Relationship between disease activity and type 1 interferon‐ and other cytokine‐inducible gene expression in blood in dermatomyositis and polymyositis. Genes Immun 2012; 13: 207–13 21881594 10.1038/gene.2011.61

[8] Salajegheh M , Kong SW , Pinkus JL , Walsh RJ , Liao A , Nazareno R , Amato AA , Krastins B , Morehouse C , Higgs BW , Jallal B . Interferon‐stimulated gene 15 (ISG15) conjugates proteins in dermatomyositis muscle with perifascicular atrophy. Ann Neurol 2010; 67: 53–63 20186858 10.1002/ana.21805 PMC2875060

[9] Preuße C , Allenbach Y , Hoffmann O , Goebel HH , Pehl D , Radke J , Doeser A , Schneider U , Alten RH , Kallinich T , Benveniste O . Differential roles of hypoxia and innate immunity in juvenile and adult dermatomyositis. Acta Neuropathol Commun 2016; 4: 45 27121733 10.1186/s40478-016-0308-5 PMC4847347

[10] Rider LG , Shah M , Mamyrova G , Huber AM , Rice MM , Targoff IN , Miller FW . The myositis autoantibody phenotypes of the juvenile idiopathic inflammatory myopathies. Medicine 2013; 92: 223–43 23877355 10.1097/MD.0b013e31829d08f9 PMC3721421

[11] Tansley SL , Betteridge ZE , Shaddick G , Gunawardena H , Arnold K , Wedderburn LR , McHugh NJ . Calcinosis in juvenile dermatomyositis is influenced by both anti‐NXP2 autoantibody status and age at disease onset. Rheumatology 2014; 53: 2204–8 24987158 10.1093/rheumatology/keu259 PMC4241891

[12] Tansley SL , Betteridge ZE , Gunawardena H , Jacques TS , Owens CM , Pilkington C , Arnold K , Yasin S , Moraitis E , Wedderburn LR , McHugh NJ . Anti‐MDA5 autoantibodies in juvenile dermatomyositis identify a distinct clinical phenotype: a prospective cohort study. Arthritis Res Ther 2014; 16: R138 24989778 10.1186/ar4600 PMC4227127

[13] Satoh M , Tanaka S , Ceribelli A , Calise SJ , Chan EK . A Comprehensive overview on myositis‐specific antibodies: new and old biomarkers in idiopathic inflammatory myopathy. Clin Rev Allergy Immunol 2017; 52: 1–19 26424665 10.1007/s12016-015-8510-y PMC5828023

[14] Tansley S , Wedderburn LR . Comparing and contrasting clinical and serological features of juvenile and adult‐onset myositis: implications for pathogenesis and outcomes. Curr Opin Rheumatol 2015; 27: 601–7 26352731 10.1097/BOR.0000000000000224

[15] Tansley SL , Simou S , Shaddick G , Betteridge ZE , Almeida B , Gunawardena H , Thomson W , Beresford MW , Midgley A , Muntoni F , Wedderburn LR . Autoantibodies in juvenile‐onset myositis: their diagnostic value and associated clinical phenotype in a large UK cohort. J Autoimmun 2017; 84: 55–64 28663002 10.1016/j.jaut.2017.06.007 PMC5656106

[16] Bohan A , Peter JB . Polymyositis and dermatomyositis (first of two parts). N Engl J Med 1975; 292: 344–7 1090839 10.1056/NEJM197502132920706

[17] Bohan A , Peter JB . Polymyositis and dermatomyositis (second of two parts). N Engl J Med 1975; 292: 403–7 1089199 10.1056/NEJM197502202920807

[18] Martin N , Krol P , Smith S , Murray K , Pilkington CA , Davidson JE , Wedderburn LR . A national registry for juvenile dermatomyositis and other paediatric idiopathic inflammatory myopathies: 10 years' experience; the Juvenile Dermatomyositis National (UK and Ireland) Cohort Biomarker Study and Repository for Idiopathic Inflammatory Myopathies. Rheumatology 2011; 50: 137–45 20823094 10.1093/rheumatology/keq261 PMC2999955

[19] Huber AM , Feldman BM , Rennebohm RM , Hicks JE , Lindsley CB , Perez MD , Zemel LS , Wallace CA , Ballinger SH , Passo MH , Reed AM . Validation and clinical significance of the Childhood Myositis Assessment Scale for assessment of muscle function in the juvenile idiopathic inflammatory myopathies. Arthritis Rheum 2004; 50: 1595–603 15146430 10.1002/art.20179

[20] Rider LG , Koziol D , Giannini EH , Jain MS , Smith MR , Whitney‐Mahoney K , Feldman BM , Wright SJ , Lindsley CB , Pachman LM , Villalba ML . Validation of manual muscle testing and a subset of eight muscles for adult and juvenile idiopathic inflammatory myopathies. Arthritis Care Res 2010; 62: 465–72 10.1002/acr.20035 PMC2924143 20391500

[21] Gunawardena H , Wedderburn LR , North J , Betteridge Z , Dunphy J , Chinoy H , Davidson JE , Cooper RG , McHugh NJ ; Juvenile Dermatomyositis Research Group UK . Clinical associations of autoantibodies to a p155/140 kDa doublet protein in juvenile dermatomyositis. Rheumatology 2008; 47: 324–8 18238791 10.1093/rheumatology/kem359

[22] Wedderburn LR , Varsani H , Li CK , Newton KR , Amato AA , Banwell B , Bove KE , Corse AM , Emslie‐Smith A , Harding B , Hoogendijk J . International consensus on a proposed score system for muscle biopsy evaluation in patients with juvenile dermatomyositis: a tool for potential use in clinical trials. Arthritis Rheum 2007; 57: 1192–201 17907237 10.1002/art.23012

[23] Varsani H , Charman SC , Li CK , Marie SK , Amato AA , Banwell B , Bove KE , Corse AM , Emslie‐Smith AM , Jacques TS , Lundberg IE . Validation of a score tool for measurement of histological severity in juvenile dermatomyositis and association with clinical severity of disease. Ann Rheum Dis 2015; 74: 204–10 24064003 10.1136/annrheumdis-2013-203396 PMC4283618

[24] Varsani H , Newton KR , Li CK , Harding B , Holton JL , Wedderburn LR . Quantification of normal range of inflammatory changes in morphologically normal pediatric muscle. Muscle Nerve 2008; 37: 259–61 17847070 10.1002/mus.20898

[25] Deakin CT , Yasin SA , Simou S , Arnold KA , Tansley SL , Betteridge ZE , McHugh NJ , Varsani H , Holton JL , Jacques TS , Pilkington CA . Muscle biopsy findings in combination with myositis‐specific autoantibodies aid prediction of outcomes in juvenile dermatomyositis. Arthritis Rheumatol 2016; 68: 2806–16 27214289 10.1002/art.39753 PMC5091622

[26] Walsh RJ , Kong SW , Yao Y , Jallal B , Kiener PA , Pinkus JL , Beggs AH , Amato AA , Greenberg SA . Type I interferon‐inducible gene expression in blood is present and reflects disease activity in dermatomyositis and polymyositis. Arthritis Rheum 2007; 56: 3784–92 17968926 10.1002/art.22928 PMC2443782

[27] Tansley SL . Antibodies in juvenile‐onset myositis. Curr Opin Rheumatol 2016; 28: 645–50 27533322 10.1097/BOR.0000000000000330

[28] Uruha A , Nishikawa A , Tsuburaya RS , Hamanaka K , Kuwana M , Watanabe Y , Suzuki S , Suzuki N , Nishino I . Sarcoplasmic MxA expression: a valuable marker of dermatomyositis. Neurology 2017; 88: 493–500 28039312 10.1212/WNL.0000000000003568

[29] Guzman J , Petty RE , Malleson PN . Monitoring disease activity in juvenile dermatomyositis: the role of von Willebrand factor and muscle enzymes. J Rheumatol 1994; 21: 739–43 8035403

[30] Shah M , Mamyrova G , Targoff IN , Huber AM , Malley JD , Rice MM , Miller FW , Rider LG . The clinical phenotypes of the juvenile idiopathic inflammatory myopathies. Medicine 2013; 92: 25–41 23263716 10.1097/MD.0b013e31827f264d PMC4580479

[31] Baechler EC , Bauer JW , Slattery CA , Ortmann WA , Espe KJ , Novitzke J , Ytterberg SR , Gregersen PK , Behrens TW , Reed AM . An interferon signature in the peripheral blood of dermatomyositis patients is associated with disease activity. Mol Med 2007; 13: 59–68 17515957 10.2119/2006-00085.Baechler PMC1869622

[32] Ekholm L , Vosslamber S , Tjärnlund A , Jong TD , Betteridge Z , McHugh N , Plestilova L , Klein M , Padyukov L , Voskuyl AE , Bultink IE . Autoantibody specificities and type I interferon pathway activation in idiopathic inflammatory myopathies. Scand J Immunol 2016; 84: 100–9 27173897 10.1111/sji.12449

[33] Pinal‐Fernandez I , Casciola‐Rosen LA , Christopher‐Stine L , Corse AM , Mammen AL . The prevalence of individual histopathologic features varies according to autoantibody status in muscle biopsies from patients with dermatomyositis. J Rheumatol 2015; 42: 1448–54 26443871 PMC6544046

[34] Allenbach Y , Leroux G , Suárez‐Calvet X , Preusse C , Gallardo E , Hervier B , Rigolet A , Hie M , Pehl D , Limal N , Hufnagl P . Dermatomyositis with or without anti‐melanoma differentiation‐ associated gene 5 antibodies: common interferon signature but distinct NOS2 expression. Am J Pathol 2016; 186: 691–700 26806087 10.1016/j.ajpath.2015.11.010

[35] Horai Y , Koga T , Fujikawa K , Takatani A , Nishino A , Nakashima Y , Suzuki T , Kawashiri SY , Iwamoto N , Ichinose K , Tamai M . Serum interferon‐alpha is a useful biomarker in patients with anti‐melanoma differentiation‐associated gene 5 (MDA5) antibody‐positive dermatomyositis. Mod Rheumatol 2015; 25: 85–9 24716595 10.3109/14397595.2014.900843

