# Supplementary material for: Expression of myxovirus‐resistance protein A: a possible marker of muscle disease activity and autoantibody specificities in juvenile dermatomyositis
Source: Neuropathol Appl Neurobiol. 2018 Jun 4;45(4):410–20. doi: 10.1111/nan.12498 (PMC6563435; doi:10.1111/nan.12498)
Supplement: Supplementary file 1 — Table S1. Clinical features and laboratory data at initial presentation between juvenile dermatomyositis (JDM) patients who had received corticosteroid and/or methotrexate and those who had no drugs prior to muscle biopsy. Table S2. Characteristics and laboratory data at initial presentation of 103 juvenile dermatomyositis (JDM) patients in different myxovirus resistance protein A (MxA) scoring data. Table S3. Distributions of patterns of myxovirus resistance protein A (MxA) staining on juvenile dermatomyositis (JDM) muscle samples across myositis‐specific autoantibody (MSA) subgroups*. [file NAN-45-410-s001.docx]

***Supplementary Table 1. Clinical features and laboratory data at initial presentation between JDM patients who had received corticosteroid and/or methotrexate and those who had no drugs prior to muscle biopsy***

| *Characteristics* | *Patients not on drugs at time of biopsy (n=92), median (IQR)** | *Patients on drugs at time of biopsy (n=11), median (IQR)** | *P-value* |
| --- | --- | --- | --- |
| *Female, n (%)* | *59 (64.1%)* | *7 (63.6%)* | *0.796* |
| *Age at disease onset, years* | *5.8 (3.8-9.2)* | *6.7 (2.6-7.7)* | *0.905* |
| *CMAS (n=90)* | *27 (14.5-45.5)* | *32 (25.5-43.5)* | *0.365* |
| *MMT8 (n=62)* | *54 (33-70.5)* | *63 (45.5-69)* | *0.563* |
| *PGA (n=72)* | *6.2 (3.6-7.9)* | *4.5 (2.9-6.4)* | *0.270* |
| *CK, units/L (n=96)* | *359 (75-3381)* | *375 (110.5-745.5)* | *0.617* |
| *hVAS* | *5 (3-7.8)* | *4 (1.5-6)* | *0.203* |
| *Total biopsy scores* | *16 (11-21)* | *14 (10-21)* | *0.516* |
| *MxA scores* | *1 (0-3)* | *0 (0-2.5)* | *0.349* |

**Data are presented as median [IQR (interquartile range)] if not stated otherwise.*

*CK, Creatinine kinase; CMAS, Childhood Myositis Assessment Scale (scores 0-52); hVAS, histopathologist’s visual analogue scale global pathology score; JDM, juvenile dermatomyositis; MMT8, Manual Muscle Testing of Eight Muscles (scores 0-80); MxA, myxovirus resistance protein A; PGA, physician global assessment (scores 0-10).*

**Supplementary Table 2. Characteristics and laboratory data at initial presentation of 103 JDM patients in different MxA scoring data**

| Variables* | MxA scores | | | | *P*-value |
| --- | --- | --- | --- | --- | --- |
|  | 0 | 1 | 2 | 3 |  |
| Female, n (%) | 26 (25.2) | 9 (8.7) | 9 (8.7) | 22 (21.4) | 0.823 |
| Age at onset, years | 8.1 (3.5-9.9) | 6.7 (4.5-10.3) | 5.1 (3.7-8.9) | 4.9 (3.5-7.1) | 0.197 |
| Time onset to biopsy, months | 5.52 (3.36-18.84) | 3.24 (2.4-4.32) | 4.08 (2.16-7.08) | 3.48 (2.04-8.52) | *0.046* |
| Nail fold changes, n (%) (n=84) | 23 (27.4) | 6 (7.1) | 7 (8.3) | 26 (30.9) | 0.200 |
| Calcinosis, n (%) (n=83) | 4 (4.8) | 1 (1.2) | 0 | 2 (2.4) | 0.091 |
| CMAS (n=90) | 41.5 (29-52) | 29 (24.5- 39) | 22 (15-26) | 19 (9-46) | *0.002* |
| MMT8 (n=62) | 68 (54-76) | 40 (32.5-55.5) | 44.5 (32-78) | 41.5 (29-63) | *0.026* |
| PGA (n=72) | 4.7 (1.7-7) | 3.6 (3-8) | 7 (3.7-7.6) | 7 (5-7.9) | 0.189 |
| CK, units/L (n=96) | 148 (54-1165) | 98 (71-301.5) | 529 (341-2510.5) | 446 (127-3702) | 0.073 |

*Data are presented as median [IQR (interquartile range)] if not stated otherwise.

CK, Creatinine kinase; CMAS, Childhood Myositis Assessment Scale (scores 0-52); MMT8, Manual Muscle Testing of Eight Muscle (scores 0-80); MxA, myxovirus resistance protein A; PGA, physician global assessment (scores 0-10).

***Supplementary Table 3. Distributions of patterns of MxA staining on JDM muscle samples across MSA subgroups*.***

| *MSA status* | *Patterns of MxA staining, n (%)* | | | |
| --- | --- | --- | --- | --- |
|  | *Perifascicular* | *Non-perifascicular* | *Both patterns* | *Negative staining* |
| *MDA5 (n=12)* | *0 (0%)* | *2 (16.7%)* | *2 (16.7%)* | *8 (66.7%)* |
| *NXP-2 (n=19)* | *6 (31.6%)* | *3 (15.8%)* | *7 (36.8%)* | *3 (15.8%)* |
| *Mi2 (n=5)* | *0 (0%)* | *2 (40%)* | *3 (60%)* | *0 (0%)* |
| *TIF1g (n=20)* | *5 (25%)* | *3 (15%)* | *7 (35%)* | *5 (25%)* |
| *No detectable (n=19)* | *2 (10.5%)* | *2 (10.5%)* | *8 (42.1%)* | *7 (36.8%)* |

**Fishers’ exact test was done to analyse the difference in the distributions and p-value was 0.084. JDM, juvenile dermatomyositis; MDA5, melanoma differentiation-associated gene 5; MSA, myositis-specific autoantibody; MxA, myxovirus resistance protein A; NXP-2, nuclear matrix protein; TIF1g, transcriptional intermediary factor 1-gamma.*
